# Children’s Eating Attitudes Test (ChEAT): a validation study in Finnish children

**DOI:** 10.1007/s40519-019-00712-w

**Published:** 2019-05-22

**Authors:** Sohvi Lommi, Heli T. Viljakainen, Elisabete Weiderpass, Rejane Augusta de Oliveira Figueiredo

**Affiliations:** 1 Folkhälsan Institute of Genetics, Folkhälsan Research Center, Helsinki, Finland; 2 Department of Food and Environmental Sciences, University of Helsinki, Helsinki, Finland; 3 Faculty of Medicine, University of Helsinki, Helsinki, Finland; 4 Department of Research, Cancer Registry of Norway, Institute of Population-Based Cancer Research, Oslo, Norway; 5 Department of Medical Epidemiology and Biostatistics, Karolinska Institutet, Stockholm, Sweden; 6 Department of Community Medicine, Faculty of Health Sciences, University of Tromsø, The Arctic University of Norway, Tromsø, Norway

**Keywords:** Adolescents, Children, Children’s Eating Attitudes Test, ChEAT, Validation, Finland

## Abstract

**Purpose:**

To validate the Children’s Eating Attitudes Test (ChEAT) in the Finnish population.

**Materials and methods:**

In total 339 children (age 10–15 years) from primary schools in Southern Finland were evaluated at two time points. They answered the ChEAT and SCOFF test questions, and had their weight, height and waist circumference measured. Retesting was performed 4–6 weeks later. Test–retest reliability was evaluated using intra-class correlation (ICC), and internal consistency was examined using Cronbach’s alpha coefficient (C-alpha). ChEAT was cross-calibrated against SCOFF and background variables. Factor analysis was performed to examine the factor structure of ChEAT.

**Results:**

The 26-item ChEAT showed high internal consistency (C-alpha 0.79), however, a 24-item ChEAT showed even better internal consistency (C-alpha 0.84) and test–retest reliability (ICC 0.794). ChEAT scores demonstrated agreement with SCOFF scores (*p* < 0.01). The mean ChEAT score was higher in overweight children than normal weight (*p* < 0.001). Exploratory factor analysis yielded four factors (concerns about weight, limiting food intake, pressure to eat, and concerns about food), explaining 57.8% of the variance.

**Conclusions:**

ChEAT is a valid and reliable tool for measuring eating attitudes in Finnish children. The 24-item ChEAT showed higher reliability than the 26-item ChEAT.

**Level of evidence:**

Level 5, cross-sectional, descriptive study.

## Introduction

Increases in the prevalence of both obesity and disordered eating are a public health concern and might be associated [1–3]. Unhealthy eating attitudes and behaviors, such as dieting, vomiting and food avoidance, are considered disordered eating symptoms (DES) [4]. Eating disorder behaviors may eventually evolve into an eating disorder (ED) or a weight disorder (overweight and obesity), and therefore is of major public health relevance [5]. ED includes anorexia nervosa, bulimia nervosa, binge eating disorder, and other specified feeding and eating disorders [6].

Among 12- to 19-year-old adolescents, the prevalence of DES may range 9–17% [7, 8], while among 12–20 year olds the prevalence of ED has been estimated around 3–4% [9–11]. Although, in general, DES and ED are more common among females than males [10, 12], the female-to-male ratio increases during puberty [13].

DES in childhood and adolescence is likely to continue into young adulthood [12, 14] and is associated with being overweight or obese, and having poor mental health and ED later in life [14–16]. Eating disturbance has been reported to start as early as the age of 9 years [17, 18]. For early recognition of DES, a reliable scale to identify DES in children is needed.

There are several instruments that can be used to assess DES in children, such as the SCOFF [19], the Eating Disorder Examination Questionnaire (EDE-Q) [20], the Eating Disorder Examination Questionnaire adapted for children (ChEDE-Q8) [21], the Eating Disorder Inventory for children (EDI-C) [22], the Kids’ Eating Disorders Survey (KEDS) [23], and the Children’s Eating Attitudes Test (ChEAT) [24]. ChEAT has been previously utilized in large cohort studies [25, 26], and it has been validated in Spain [27, 28], Portugal [29], Belgium [30], Japan [31], and the USA [24, 32, 33]. ChEAT has also been successfully used in Sweden [34]; however, to our knowledge, it has not yet been validated in Finland or in any of the other Nordic countries.

The aim of the present study is to validate ChEAT in a community sample of 10- to 15-year-old Finnish school children. This study is a part of the Finnish Health in Teens (Fin-HIT) cohort study.

## Methods

### Participants

**Fig. 1:**
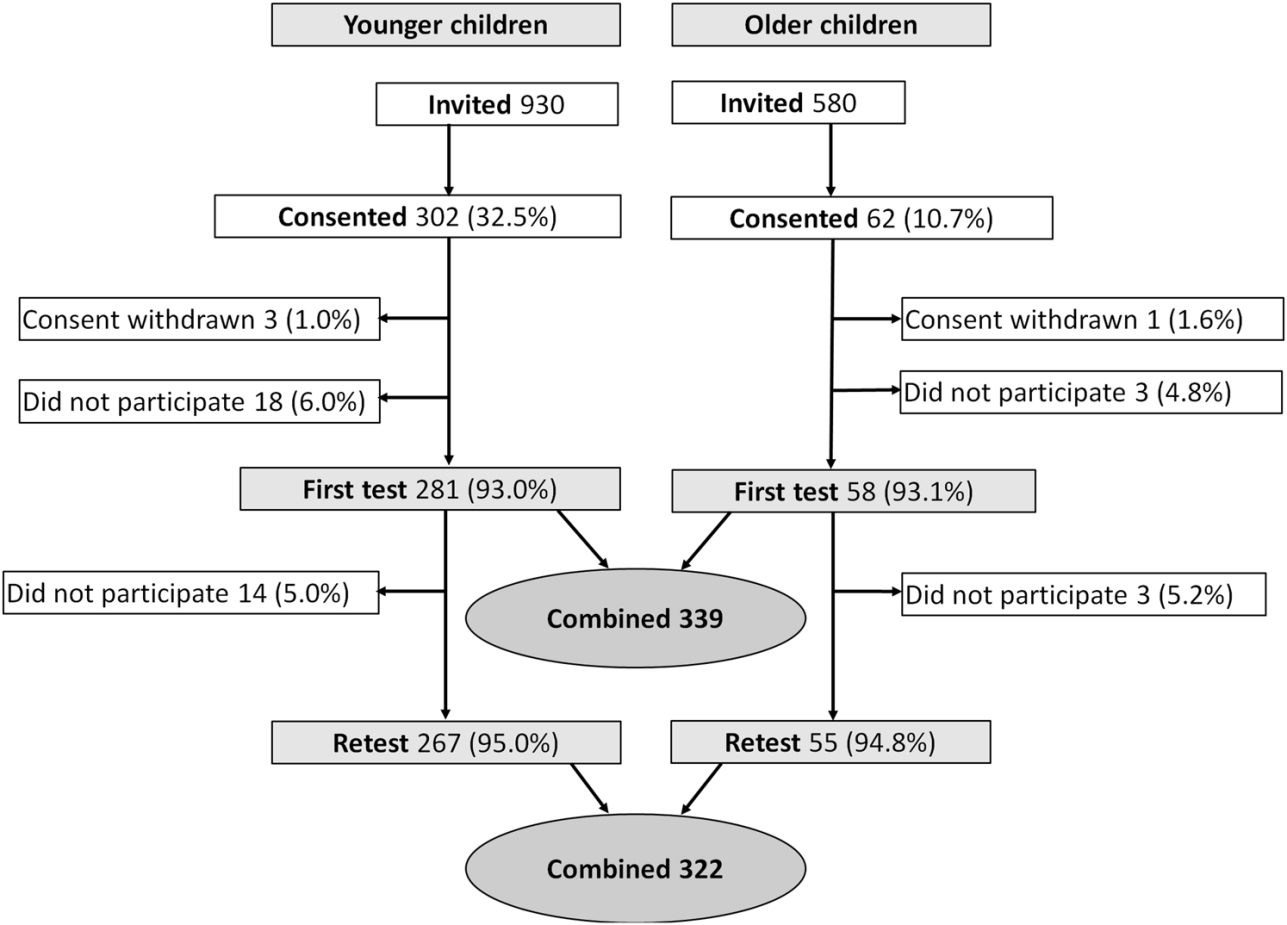
Flowchart of study

The present study, conducted by Folkhälsan Research Center in Helsinki, was initiated to validate ChEAT among Finnish children. ChEAT was used in the Fin-HIT cohort to evaluate disordered eating symptoms [35], and it was chosen as it had been successfully validated in several countries, as described above, and utilized in several previous studies (e.g., [17, 34, 36]). The Fin-HIT is a prospective cohort with approximately 11,000 9- to 14-year-old participants across Finland. The proposed validation sub-study consisted of 339 children at the same age as children in the Fin-HIT cohort at baseline. Figure 1 shows the participant selection flowchart. Participants were recruited from 12 primary schools in the Helsinki metropolitan area, and the data collection was conducted between October 2017 and March 2018. After receiving recruitment permission from municipal education administration and the principal of each school, fieldworkers introduced the study protocol and distributed invitations to children in suitable classes. To participate, children and one parent per each child provided a written informed consent. The Coordinating Ethics Committee of the Hospital District of Helsinki and Uusimaa has approved the study protocol.

### Data collection

The data collection was conducted at two time points during normal school days: an initial visit and a retest visit 4–6 weeks later. Participants answered a questionnaire consisting of ChEAT, a question related to overeating symptoms (frequency of having eaten too much within a short time) [37], SCOFF and a short food frequency questionnaire. Instructions to fill in the questionnaire were explained simultaneously to all participants, and possible questions arising afterward were answered individually. After completing the questionnaire, at the first visit only, participants’ weight, height and waist circumference were measured by trained fieldworkers. Due to absence from school, some participants filled in the questionnaire and took their measurements at home. We have previously shown the validity of home-based measures of weight, height, and waist circumference, and these were found to be reliable [38]. Of the 339 participants who attended the first data collection, 6 participated at home, and of the 322 retested participants, 16 participated at home.

### Study sample size

Sample size calculations were based on ChEAT scores obtained from children in the Fin-HIT cohort (mean 6.1, standard deviation 4.7) [25]. Adopting a significance level of 5% and a power of 80%, the minimum required sample size calculated was 214 children, allowing us to compare the results between the two time points (test and retest) and to test the internal consistency of the scale. We recruited children in 5th to 8th grades, but since the response rate was low among children in 7th and 8th grades (hereafter referred to as older children), we chose to carry out the study with children in 5th and 6th grades (hereafter referred to as younger children). The adequate sample size was met with younger children. The 58 older children were included as a complementary sample.

### Measures

#### ChEAT

ChEAT is a self-administered questionnaire for assessing eating attitudes and behaviors in children, based on the Eating Attitudes Test (EAT-26) used in adults [39]. Since some wordings of EAT-26 items were deemed incomprehensible to children, a simplified version was developed and validated [24]. ChEAT consists of 26 items scored on a 6-point Likert scale with answer options ‘never’, ‘rarely’, ‘sometimes’, ‘often’, ‘usually’, and ‘always’. To calculate the scale’s scores, the categories ‘never’, ‘rarely’, and ‘sometimes’ were scored as zero, ‘often’ as one, ‘usually’ as two, and ‘always’ as three. Item 19 was reverse-scored, as suggested by Maloney [24]. The maximum score is 78 points. The higher the score, the higher the possibility for DES. The original ChEAT had three subscales: “Dieting”, “Bulimia and Food Preoccupation” and “Oral Control”, respectively, with 13, 6, and 7 items [39]. ChEAT was created and validated in English. The instrument used in Fin-HIT cohort and in the present study was evaluated by the research group and the final version was translated into Finnish by linguistic professionals. The scale adaptation for Finnish-speaking children was performed at the beginning of the Fin-HIT study.

#### SCOFF

The questionnaire also included SCOFF, a screening tool for detecting DES, consisting of five items that are dichotomously scored (yes = 1, no = 0). Two or more “yes” responses indicate DES [19]. The acronym SCOFF is composed of the following questions: (1) do you make yourself **S**ick because you feel uncomfortably full?; (2) do you worry you have lost Control over how much you eat?; (3) have you recently lost more than 6 kg (one stone) in a 3-month period?; (4) do you believe yourself to be Fat when others say you are too thin?; and (5) would you say that Food dominates your life? SCOFF was used to assess the concurrent validity since it was validated and used in several Finnish studies [40, 41].

#### Anthropometric measurements

Weight, height and waist circumference were measured by field workers in a standardized way. Weight was measured to the nearest 0.01 kg with a portable digital scale (CAS model PB), and height to the nearest 0.1 cm with a portable stadiometer (Seca model 217). Digital scale was calibrated daily before each series of measurements. Before measurements, the participants removed their shoes, heavy sweaters, and any objects from their pockets. Fieldworkers reported the clothing participants were wearing, and the weight of the clothes was subtracted from the measured weight in a standardized way. Waist circumference was measured midway between the hip bones and ribs to the nearest 0.1 cm with a measuring tape calibrated against a measure approximately once a week. Waist measurement was repeated twice, and the recorded waist circumference was calculated as their mean. In cases with more than 1 cm difference between the two measurements, a third measurement was performed and the mean was calculated based on all three measurements.

Body mass index (BMI) was calculated as weight (kg)/height (m)^2^, and the children were classified as underweight, normal weight, overweight, or obese according to age- and sex-specific cut-offs suggested by the International Obesity Task Force (IOTF) [42]. Information on age and gender was obtained from the consent form and the questionnaire.

### Statistical analysis

All associations between categorical variables were assessed using Chi-square tests. Comparison of quantitative variables with dichotomous variables was carried out using Student’s *t* test. ChEAT was evaluated for 300 children who answered all ChEAT items. Comparisons between ChEAT score and BMI categories were performed by generalized linear models using Poison distribution. The internal consistency of all scale items was assessed using Cronbach’s alpha coefficient (C-alpha), evaluating the correlation of each item with all the other items together.

To evaluate the factorial structure of all items and to identify different subscales in ChEAT, we used exploratory factor analysis. A model’s goodness-of-fit was evaluated by the Kaiser–Meyer–Olkin (KMO) and Bartlett’s sphericity tests, considering values over 0.70 and *p* < 0.05, respectively, as acceptable [43]. Once the factors were identified, confirmatory factor analysis (CFA) was used to check the scale’s factor structures. Estimations for CFAs were carried out using a robust unweighted least squares method. The goodness-of-fit of these two models was assessed using the following measures: Chi-square test, comparative fit index (CFI), non-normed fit index (NNFI), root mean square error (RMSEA), standardized mean square residual (SRMR), goodness-of-fit index (GFI), and adjusted goodness-of-fit index (AGFI) [44].

Test–retest results were evaluated using paired t tests to compare mean scores, Pearson correlation and intra-class correlation (ICC) to evaluate reliability and homogeneity of results between the two time points.

We used the Lavaan package in R for CFA and SPSS statistical software (version 24.0) for all other statistical analyses. We adopted a 5% statistical significance level for all tests.

## Results

### Descriptive

Of the 339 participants, 82.9% (*n* = 281) were younger and 17.1% (*n* = 58) older children. The overall mean age of participants was 11.9 (SD ± 0.9) years: 11.6 (± 0.6) years for younger and 13.3 (± 0.7) years for older children. Overall, 54.6% (*n* = 185) were girls and 45.4% (*n* = 154) were boys. Table 1 shows the main characteristics of all participants, and comparisons between younger and older children. Gender, BMI and presence of disordered eating did not differ between younger and older children.

**Table 1:** Characteristics of all children and separated by age groups

	All children		Age group				*p* value
			Younger children		Older children		
	*n*	%	*n*	%	*n*	%	
Gender							
Girls	185	54.6	155	55.2	30	51.7	0.632
Boys	154	45.4	126	44.8	28	48.3	
BMI							
Underweight	41	12.1	36	12.8	5	8.6	0.664
Normal weight	246	72.6	201	71.5	45	77.6	
Overweight	49	14.5	41	14.6	8	13.8	
Obese	3	0.9	3	1.1	0	0.0	
School size							
< 300 students	91	26.8	91	32.4	0	0.0	< 0.001
> 300 students	248	73.2	190	67.6	58	100.0	
DES (SCOFF ≥ 2)							
No DES	284	89.3	232	88.2	52	94.5	0.167
DES	34	10.7	31	1.8	3	5.5	

	Mean	SD	Mean	SD	Mean	SD	
ChEAT score (26 items)	2.55	4.22	2.66	4.44	2.05	2.84	0.319
Age (years)	11.9	0.9	11.6	0.6	13.3	0.7	< 0.001
Waist circumference (cm)	65.4	7.4	64.6	7.2	69.3	7.1	< 0.001
Weight (kg)	44.6	10.3	42.8	9.1	53.0	11.6	< 0.001

*BMI* body mass index, *DES* disordered eating symptoms, *SD* standard deviation

### Internal consistency

Cronbach’s alpha coefficient (C-alpha) showed high internal consistency (C-alpha 0.79) for the 26-item ChEAT, however, the reverse-scored item 19 (I can show self-control around food) and the item 25 (I enjoy trying new rich foods) had a negative correlation with other items. A negative correlation indicates a reverse direction of these items related to the other items, suggesting that they should not be included in the final scale (called the 24-item ChEAT). After exclusion of the two items, the internal consistency increased (C-alpha  0.84). The internal consistency was also evaluated separately for younger and older children, and there was improvement in the C-alpha after exclusion in both groups: increasing from 0.81 to 0.84 in younger children, and from 0.64 to 0.73 in older children.

### Test and retest

Table 2 shows results for test and retest analysis. We found no statistical difference between test and retest in the mean total ChEAT scores, either with the 26-item ChEAT (*p* = 0.331) or the 24-item ChEAT (*p* = 0.119). The results remained similar when stratified by age group. When evaluating the homogeneity between the two time points using ICC, the overall homogeneity was higher with the 24-item ChEAT (ICC 0.794) than with the 26-item ChEAT (ICC 0.769). Similar results were obtained when separated by age group (Table 2).

**Table 2:** Results of the test and retest analysis for all children and separated by age groups

	ChEAT score (test)		ChEAT score (retest)		Paired *t* test (*p* value)	Pearson correlation	ICC
	Mean	SD	Mean	SD			
26-item ChEAT							
All children	2.55	4.22	2.40	3.91	0.331	0.771	0.769
Younger children	2.66	4.44	2.45	4.10	0.266	0.780	0.779
Older children	2.05	2.84	2.16	2.83	0.774	0.680	0.670
24-item ChEAT							
All children	2.03	4.13	1.80	3.81	0.119	0.796	0.794
Younger children	2.12	4.37	1.86	3.99	0.129	0.797	0.795
Older children	1.57	2.73	1.53	2.83	0.717	0.784	0.784

*ICC* intra-class correlation, *SD* standard deviation

### Cross-calibrating ChEAT against SCOFF and other variables

We compared the mean score of ChEAT with the mean score of SCOFF and with the presence of the overeating symptom (frequency of having eaten too much in a short time) using the t test (Table 3). We found that the mean ChEAT score is higher in children with DES based on SCOFF than in children without DES (*p* < 0.01). Similar results were observed among the different age groups. Children with overeating at least once a month had higher ChEAT scores than children without overeating (*p* = 0.013). We obtained the same results among younger children (*p* = 0.009), but not with older children (*p* = 0.769).

**Table 3:** Comparison of ChEAT score by SCOFF score, binge eating symptom, gender, and body mass index (BMI) categories for all children and separated by age groups

	All			*p* value	Younger			*p* value	Older			*p* value
	*n*	Mean	SD		*n*	Mean	SD		*n*	Mean	SD	
26-item ChEAT												
SCOFF												
No DES	246	1.85	2.58	< 0.001	198	1.87	2.58	0.010	48	1.79	2.58	0.005
DES	31	6.94	9.29		28	6.96	9.67		3	6.67	5.51	
Frequency of having eaten too much in short time												
Never	212	1.94	2.94	0.013	174	1.90	2.93	0.009	38	2.13	3.00	0.769
Sometimes in a month	79	3.68	5.89		64	4.11	6.34		15	1.87	2.80	
Gender												
Girls	163	2.88	5.04	0.061	134	3.06	5.39	0.051	29	2.07	2.83	0.971
Boys	135	1.99	3.05		110	1.98	3.07		25	2.04	3.02	
BMI												
Underweight	36	2.00	2.06	< 0.001^a^	32	2.16	2.08	< 0.001^a^	4	0.75	1.50	0.150
Normal	218	2.31	3.41		176	2.36	3.56		42	2.10	2.68	
Overweight/obese	44	3.70	7.88		36	3.97	8.49		8	2.50	4.38	
24-item ChEAT												
SCOFF												
No DES	284	1.47	2.50	0.004	232	1.50	2.54	0.009	52	1.33	2.34	0.001
DES	34	6.29	9.14		31	6.26	9.48		3	6.67	5.51	
Frequency of having eaten too much in short time												
Never	243	1.52	2.90	0.009	202	1.52	2.92	0.008	41	1.54	2.81	0.919
Sometimes in a month	88	3.22	5.69		72	3.57	6.12		16	1.63	2.68	
Gender												
Girls	185	2.27	4.83	0.235	155	2.43	5.12	0.186	30	1.43	2.79	0.999
Boys	154	1.73	3.10		126	1.74	3.19		28	1.71	2.71	
BMI												
Underweight	41	1.32	2.13	< 0.001^b^	36	1.50	2.21	< 0.001^a^	5	0.00	0.00	0.069
Normal	246	1.80	3.22		201	1.85	3.36		45	1.58	2.48	
Overweight/obese^a^	52	3.67	7.52		44	3.89	7.98		8	2.50	4.38	

*SD* standard deviation, *DES* disordered eating symptoms, *BMI* body mass index

^a^Multiple comparison between overweight and other groups *p* < 0.001

^b^Multiple comparison between all BMI categories *p* < 0.05

Mean ChEAT scores were also compared between genders and BMI groups. The mean ChEAT score did not differ between genders (*p* = 0.061), but did differ between BMI categories (*p* < 0.001): higher scores were related with higher BMI. Similar results were obtained when using the 24-item ChEAT score (Table 3).

### Exploratory and confirmatory factor analysis

After excluding the two items from the scale, we performed a factor analysis to evaluate the factorial structure. Factor analysis identified four subscales (i.e., four factors; Table 4) with high adaptability to the original data (KMO = 0.775 and *p* < 0.001 for Bartlett’s sphericity test), and these explained 57.8% of the data variability.

**Table 4:** Loads for new 24-item ChEAT factors after exploratory factor analysis

	Factor 1	Factor 2	Factor 3	Factor 4
Item 1	0.494	0.073	0.191	0.036
Item 6	0.438	− 0.075	0.347	0.202
Item 7	0.614	0.343	0.064	0.160
Item 10	0.814	− 0.025	0.182	− 0.063
Item 11	0.630	0.329	0.117	− 0.061
Item 12	0.534	0.148	0.242	0.165
Item 13	0.307	− 0.005	0.182	0.091
Item 14	0.786	− 0.054	− 0.032	− 0.089
Item 17	0.600	0.114	− 0.106	0.426
Item 18	0.421	0.089	0.017	0.165
Item 22	0.427	− 0.052	− 0.020	0.231
Item 23	0.542	0.187	− 0.055	0.137
Item 24	0.316	0.195	− 0.046	0.151
Item 2	0.141	0.586	0.424	0.100
Item 4	0.418	0.660	− 0.043	0.270
Item 5	0.013	0.607	0.069	− 0.120
Item 9	0.034	0.642	− 0.081	0.365
Item 26	0.048	0.894	0.081	− 0.077
Item 8	0.001	0.020	0.526	− 0.089
Item 15	0.178	0.276	0.451	− 0.086
Item 20	0.380	0.364	0.455	0.149
Item 21	− 0.023	− 0.051	0.748	0.258
Item 3	0.092	0.061	0.024	0.715
Item 16	0.159	0.011	0.096	0.332

We used CFA to verify the scale structure with three different approaches: (1) a model with the original structure; (2) a model with the original structure excluding items 19 and 25; and (3) a model with a new structure with four factors as suggested by the exploratory factor analysis (Table 5). In all children, model 1 showed the poorest goodness-of-fit compared with models 2 and 3 for all measures. Both model 2 and 3 showed high goodness-of-fit in all children, according to the following measures: CFI, NNFI, RMSEA, Chi-square, GFI and AGFI [44]. Similar results were obtained in the subgroup of younger children. The sample size was not large enough to evaluate models separately in the older children. After checking the goodness-of-fit of model 3, we evaluated the standard factor loads estimated by the CFA (Table 6), interpreted the results and named the four factors accordingly: subscale 1 as “Concerns about weight”; subscale 2 as “Limiting food intake”; subscale 3 as “Pressure to eat”; and subscale 4 as “Concerns about food”. To cross-validate our results, we carried out the CFA with 11,407 children from Fin-HIT cohort, and found similar results: even though the results for the 26-item scale (model 1) were good, the results for the 24-item scale were even better (model 2 and 3) (Table 5).

**Table 5:** Results of confirmatory factorial analysis for three different models evaluated in the study for all children and younger children

	Model 1	Model 2	Model 3	Criteria [44]
	(26 items—original)	(24 items—original)	(24 items—4 new factors)	
All children				
CFI	0.872	0.959	0.955	CFI ≥ 0.90
NNFI	0.859	0.953	0.948	NNFI ≥ 0.90
RMSEA	0.028	0.016	0.017	RMSEA < 0.08
SRMR	0.101	0.102	0.091	SRMR < 0.08
Chi-square	0.003	0.167	0.144	*p* value > 0.05
GFI	0.976	0.974	0.971	GFI ≥ 0.95
AGFI	0.970	0.966	0.963	AGFI ≥ 0.90
Younger children				
CFI	0.842	0.941	0.959	CFI ≥ 0.90
NNFI	0.826	0.932	0.953	NNFI ≥ 0.90
RMSEA	0.032	0.021	0.017	RMSEA < 0.08
SRMR	0.112	0.113	0.102	SRMR < 0.08
Chi-square	0.001	0.110	0.194	*p* value > 0.05
GFI	0.970	0.971	0.966	GFI ≥ 0.95
AGFI	0.962	0.960	0.957	AGFI ≥ 0.90
Cross-validation analysis—using Fin-HIT data (*n* = 11,407)				
CFI	0.950	0.969	0.991	CFI ≥ 0.90
NNFI	0.891	0.965	0.944	NNFI ≥ 0.90
RMSEA	0.041	0.043	0.038	RMSEA < 0.08
SRMR	0.054	0.056	0.057	SRMR < 0.08
Chi-square	0.098	0.076	0.072	*p* value > 0.05
GFI	0.990	0.980	0.986	GFI ≥ 0.95
AGFI	0.989	0.976	0.982	AGFI ≥ 0.90

*CFI* comparative fit index, *NNFI* non-normed fit index, *RMSEA* root mean square error, *SRMR* standardized mean square residual, *GFI* goodness-of-fit index, *AGFI* adjusted goodness-of-fit index

**Table 6:** Confirmatory factorial analysis results for 24-item ChEAT

Item	Standardized estimation
Subscale 1 “concerns about weight”	
1—I am scared about being overweight	0.451
6—I am aware of the energy (calorie) content in foods that I eat	0.538
7—I try to stay away from foods such as breads, potatoes, and rice	0.536
10—I feel very guilty after eating	0.703
11—I think a lot about wanting to be thinner	0.615
12—I think about burning up energy (calories) when I exercise	0.664
13—Other people think I am too thin	0.310
14—I think a lot about having fat on my body	0.624
17—I eat diet foods	0.558
18—I think that food controls my life	0.398
22—I feel uncomfortable after eating sweets	0.435
23—I have been dieting	0.517
24—I like my stomach to be empty	0.348
Subscale 2 “limiting food intake”	
2—I stay away from eating when I am hungry	0.800
4—I have gone on eating binges where I feel that I might not be able to stop	0.690
5—I cut my food into small pieces	0.389
9—I vomit after I have eaten	0.483
26—I feel I have to vomit after a meal	0.604
Subscale 3 “pressure to eat”	
8—I feel that others would like me to eat more	0.237
15—I take longer than others to eat my meals	0.508
20—I feel that others pressure me to eat	0.824
21—I give too much time and thought to food	0.395
Subscale 4 “concerns about food”	
3—I think about food a lot of the time	0.428
16—I stay away from foods with sugar in them	0.503

## Discussion

The present study validated the ChEAT scale in a population of Finnish children and proposed a new 24-item ChEAT. We identified new subscales to evaluate different aspects of DES in children. Validation was conducted with different approaches, all showing good consistency and reliability.

We evaluated the internal consistency of the original 26-item scale and observed two items (19 ‘I can show self-control around food’ and 25 ‘I enjoy trying new rich foods’) having an inverse correlation with all other items, indicating a semantic inversion of the statements, thus suggesting that these items were not clear for all children. The inverse correlation of the reverse-scored item 19 could be due to response bias, since the participants may have had a tendency to reply to this item in the same direction as to the other items. By excluding these two items, the reliability of the scale improved. The CFA reinforced these findings, since a better goodness-of-fit was observed with the 24-item scale. The exclusion of one or both of these items have been suggested by others too [24, 30, 31, 33], resulting in improved consistency.

We cross-calibrated ChEAT with other variables that have been associated with DES in previous studies. The SCOFF is a widely used, validated screening tool in clinical settings in Finland [41, 45, 46]. We observed that the mean ChEAT score was higher among those who scored higher in SCOFF. The ChEAT also discriminated between children with overeating and without overeating. Previous studies show that overweight children are more likely to have DES [47, 48], and our result of overweight children having higher ChEAT scores than normal weight children was in line with these. We also compared ChEAT score by gender but did not find any difference, which is consistent with results from some earlier studies [28, 49]. However, DES is shown to be more frequent in girls than boys in some studies [50, 51], but the gender difference is proposed to emerge around the age of 13 [52–54].

The mean ChEAT scores in our sample were lower than observed in previous studies [31, 33, 55]. The majority of our participants lived in a small or medium-sized city or in the rural areas of a large city, which could partly explain our findings since mean ChEAT scores have been observed to be higher in large cities than in small and medium-sized cities, possibly due to susceptibility to more social pressure and to emphasized media influence in larger cities [31]. It has been suggested that socioeconomic status affects the prevalence of DES [56, 57], which might explain low mean scores in our study. However, information on parental SES was not available here.

Our exploratory factor analysis yielded four factors or subscales that we named “Concerns about weight”, “Limiting food intake”, “Pressure to eat”, and “Concerns about food” that describe relevant attitudes and behaviors. The factor structure was confirmed in the CFA. In previous studies, the typical number of factors has been four or five, which is in line with our results [27, 31, 55, 58–60]. In some previous studies [27, 31, 61], factor analysis revealed a factor representing behaviors or thoughts related to purging. In our analysis, purging (‘I vomit after I have eaten’ and ‘I have the urge to vomit after a meal’) did not stand out as an independent factor, but rather was associated with behaviors related to controlling the amount of food eaten.

Typically, validation studies are performed without proper sample size calculations [62], but this study was performed with an adequate sample size. We used several statistical approaches to validate ChEAT. Complementarily, we cross-calibrated ChEAT against several background variables that included SCOFF, overeating symptom, BMI, and gender. Furthermore, a cross-validation analysis in 11,407 Fin-HIT participants confirmed the results of the CFA. Still, this study had some limitations. The low participation rate may cause some bias. The participants are likely to be more health-oriented than the non-participants [63, 64], which could be the reason for low mean ChEAT scores in general. The low participation rate might also be due to a lack of a systematic reminder to parents to participate. Teachers were asked to send a reminder to parents through a messaging system, but it was not ensured whether they actually sent them. Since we did not have ED diagnoses available, we were not able to evaluate a ChEAT cut-off for identifying DES. The number of studies investigating a suitable cut-off for children is limited [27, 28, 31]. Cut-offs varying from 10 to 20 have been tested in age groups between 9 and 17 [27, 28, 31], but there is yet no consensus on an optimal cut-off. The Fin-HIT cohort will include data on ED diagnoses from national healthcare registers, thus it will be possible to determine a suitable cut-off for children in the future. Moreover, the restricted range of the ChEAT scoring could be a possible limitation in our study. However, the restricted range reduces correlation coefficient values [65], which in this study is not a problem as we yielded good correlation coefficients even with the restricted range.

To conclude, we assessed the consistency and reliability of the ChEAT using an adequate sample size among Finnish children. ChEAT is a valid and reliable tool to measure eating attitudes and behaviors in Finnish children in 5th and 6th grades (10–13 years). We proposed a 24-item ChEAT to be used in future studies among Finnish children.
